# Droplet motion driven by humidity gradients during evaporation and condensation

**DOI:** 10.1140/epje/s10189-024-00426-7

**Published:** 2024-05-13

**Authors:** Hernán Barrio-Zhang, Élfego Ruiz-Gutiérrez, Daniel Orejon, Gary G. Wells, Rodrigo Ledesma-Aguilar

**Affiliations:** 1 Institute for Multiscale Thermofluids, School of Engineering, University of Edinburgh, The King’s Buildings, Mayfield Road, Edinburgh, EH9 3FB UK; 2 School of Engineering, Newcastle University, Claremont Road, Newcastle upon Tyne, NE1 7RU UK

## Abstract

**Abstract:**

The motion of droplets on solid surfaces in response to an external gradient is a fundamental problem with a broad range of applications, including water harvesting, heat exchange, mixing and printing. Here we study the motion of droplets driven by a humidity gradient, i.e. a variation in concentration of their own vapour in the surrounding gas phase. Using lattice-Boltzmann simulations of a diffuse-interface hydrodynamic model to account for the liquid and gas phases, we demonstrate that the droplet migrates towards the region of higher vapour concentration. This effect holds in situations where the ambient gradient drives either the evaporation or the condensation of the droplet, or both simultaneously. We identify two main mechanisms responsible for the observed motion: a difference in surface wettability, which we measure in terms of the Young stress, and a variation in surface tension, which drives a Marangoni flow. Our results are relevant in advancing our knowledge of the interplay between gas and liquid phases out of thermodynamic equilibrium, as well as for applications involving the control of droplet motion.

**Graphic abstract:**

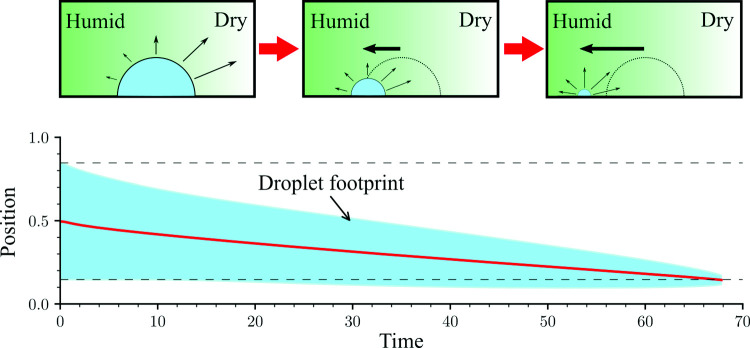

**Supplementary Information:**

The online version contains supplementary material available at 10.1140/epje/s10189-024-00426-7.

## Introduction

The controlled motion of a liquid droplet on a solid surface is important in many applications, including water harvesting [1, 2], heat exchange [3], microreactors [4] and printing [5]. The driving forces responsible for droplet motion comprise capillary forces which include the interaction with the solid, the wettability, and those which result from variations of the droplet’s surface tension, also known as Marangoni forces [6, 7]. Wetting dominated motion can be achieved by introducing a gradient in the solid surface’s properties, e.g. its chemical composition [8], elasticity [9], or topography [10]. On the other hand, when differences in the surface tension along the droplet’s surface arise, these drive Marangoni flows towards the region of higher surface tension, which can in turn be strong enough to drive the motion of the droplet. Such an effect can be achieved through variations in composition or temperature along the droplet’s surface [11, 12].

Achieving droplet motion through variations in the composition of the ambient gas phase can be advantageous, as such a strategy eliminates the need for patterning the solid surface and allows dynamic control. It is well known that the interactions between pairs of droplets mediated by the surrounding gas phase can generate motion of the droplet.

In general, one can consider two cases for the droplet-gas interactions: composite systems, where droplets are made of different liquids or liquid mixtures [13–16], and pure systems [17–19], where both droplets are made of the same substance. Droplet motion in composite systems has been shown to be driven by Marangoni flows, which arise from a varying composition at the droplet surface driven by a different volatility and surface tension of the components of the droplet [13].

The motion of droplets driven by variations of the concentration of the droplet’s own vapour in the ambient gas phase, i.e. its *humidity*, is less well understood. In 2017, Man and Doi [19] performed a theoretical study of droplets evaporating on a smooth, chemically homogeneous solid surface using a one-sided model based on lubrication theory. For pairs of single-component droplets, they studied the effect of an imposed variation of the evaporative flux across the droplet’s interface and predicted droplet motion towards regions of lower evaporative flux. This prediction was confirmed experimentally by Wen et al. [17]. In further experiments, Sadafi et al. [18] reported that the mechanism leading to motion can also be influenced by Marangoni flows arising from a variation in temperature due to evaporative cooling.

The liquid-vapour phase change of sessile droplets is a complex phenomenon that involves mass and heat transfer [20, 21]. While progress has been made in understanding the mechanism leading to motion of droplets undergoing evaporation, a study of the fluid dynamics in the liquid and gas phases is still missing. Furthermore, whether motion can also arise during droplet condensation, is still an open question. Therefore, in this work we perform a computational study of the mechanism leading to droplet motion of a single-component droplet due to variations of the concentration of its vapour in the ambient gas phase and consider both evaporation and condensation of droplets. To isolate this effect, we neglect other effects such as temperature gradients or concentration gradients at the liquid–gas interface. We use lattice-Boltzmann simulations of a binary-fluid hydrodynamic model, which has been validated previously in studies of droplet evaporation under isothermal conditions [22].

In Sect. 2 we present the model equations and the lattice-Boltzmann simulation setup. In Sect. 3 we report the results of our work. We start by presenting results of droplet motion under a gradient in the ambient fluid phase composition and then analyse the variation of the surface tension along the droplet’s surface and the difference in the Young’s stress at the contact line. We show that the gradient in the composition of the ambient phase leads to a variation of the mass flux along the interface as predicted by Man and Doi [19]. However, we also identify two competing effects in the simulations: a variation of the surface tension along the droplet’s surface, which induces flow towards regions of lower concentration of vapour in the gas phase, and a difference in Young’s stresses at the contact line, which drives the droplet towards regions of higher vapour concentration. Finally, in Sect. 4 we present the conclusions of this work.

## Methodology

### Governing equations

**Fig. 1:**
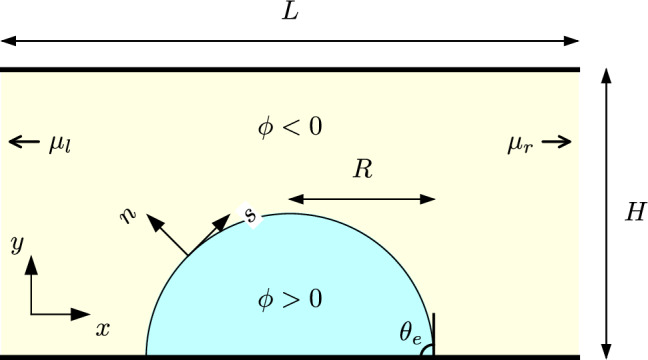
Schematic representation of the system. A two-dimensional droplet of base radius *R* and equilibrium contact angle $\theta _e$ sits on the bottom solid surface of a rectangular channel of length *L* and height *H*. The droplet and the surrounded gas phase are distinguished by an order-parameter field, $\phi$. A gradient in the composition of the gas phase is induced by fixing the chemical potential at the left and right open edges of the channel, to values $\mu _l$ and $\mu _r$, respectively. *n* and *s* represent the normal and tangential vector with respect of the interface of the droplet, respectively, and define the direction of the arc-length

Figure 1 shows a schematic representation of the system. We consider a rectangular domain of length *L* and height *H*. The top and bottom edges, located at $y=0$ and $y=H$, correspond to flat solid surfaces. The left and right edges, where $x=0$ and $x=L$, are open boundaries. The liquid droplet sits on the bottom solid surface and is surrounded by a gas phase, which spans the rest of the domain.

To model the liquid droplet and the surrounding gas, we consider a binary-fluid model able to exhibit the coexistence of two phases: a liquid-rich phase, corresponding to the droplet, and a liquid-poor phase, corresponding to the surrounding gas. Following a mesoscopic approximation, the two phases are described by a continuous-order parameter, $\phi (\textbf{r},t)$, which varies smoothly from one phase to the other across a diffuse interfacial region. The region occupied by the fluids is denoted by *V* and the solid boundary by *S*. A suitable choice of the Helmholtz free energy of such a system is given by [23]

1 $$\begin{aligned} \mathcal {F}[\rho , \phi ] \!\!= \!\!\int _{V} \left[ \psi (\phi , \nabla \phi ) \!\!+ \!\!\frac{1}{3}\rho \log \rho \right] \,\textrm{d}V \!\!+\!\! \int _{S} \zeta (\phi ) \textrm{d}S, \end{aligned}$$

The first integral in Eq. (1) is the bulk contribution to the free energy. The first term in the integrand is the interaction energy, defined as

2 $$\begin{aligned} \psi (\phi , \nabla \phi ) = \frac{3\gamma _{\textrm{eq}}}{\sqrt{8}l}\bigg (\frac{1}{4}\phi ^4 - \frac{1}{2}\phi ^2 + \frac{1}{2}l^2|\nabla \phi |^2\bigg ). \end{aligned}$$

Here, the $\phi ^2$ and $\phi ^4$ terms produce a double-well potential with minima at $\phi = \pm \phi _{\textrm{eq}}$, which correspond to the saturation values of the order parameter in the liquid and gas for a flat interface configuration. Here, the model parameters are chosen so that $\phi _{\textrm{eq}} = 1$. Therefore, we will take $\phi > 0$ as the droplet and $\phi < 0$ as the surrounding gas, thus defining the curve $\phi (\textbf{r}) = 0$ as the droplet–gas interface. The square-gradient term in Eq. (2) gives rise to an energy cost from one phase to the other, therefore producing a smooth/diffuse interface of equilibrium surface tension $\gamma _{\textrm{eq}}$ and thickness *l*, respectively. The second term in the first integrand of Eq. (1) corresponds to an ideal-gas contribution due to the kinetic motion of molecules in the fluid, where $\rho$ is the mass density.

The second integral in Eq. (1) represents the free-energy contribution from the interaction of the fluid and the solid surface, where $\zeta (\phi )$ can be used to control the wettability of the solid. Here we use $\zeta (\phi )= - \chi \phi$, which leads to the natural boundary condition

3 $$\begin{aligned} \frac{\partial \phi }{\partial n}=-\frac{\sqrt{8}\chi }{3\gamma _{\textrm{eq}} l}, \end{aligned}$$

where *n* denotes the direction normal to the solid surface. It can be shown that the equilibrium contact angle, $\theta _e$, obeys

4 $$\begin{aligned} \chi = \frac{3}{2} \gamma _{\textrm{eq}} \textrm{sgn}\left( \frac{\pi }{2}-\theta _e\right) \sqrt{\cos \varphi (1-\cos \varphi )}, \end{aligned}$$

where $$\varphi =\frac{1}{3}\arccos {(\sin ^2\theta _e)}$$.

The local chemical potential of the fluid is defined as

5 $$\begin{aligned} \mu (\phi ) \equiv \frac{\delta \mathcal F}{\delta \phi } = \frac{3\gamma _{\textrm{eq}}}{\sqrt{8}l} \left( \phi ^3 - \phi - l^2 \nabla ^2 \phi \right) \end{aligned}$$

In equilibrium, $\mu = 0$. Out of equilibrium, inhomogeneities in the chemical potential lead to a diffusive flux

6 $$\begin{aligned} \textbf{J} = -M\nabla \mu , \end{aligned}$$

where *M*, called the mobility, plays the role of the diffusivity. Imposing the conservation of the order parameter leads to the Cahn–Hilliard convection-diffusion equation [24],

7 $$\begin{aligned} \partial _t\phi + \nabla \cdot ({\textbf {u}}\phi ) = -\nabla \cdot \textbf{J}, \end{aligned}$$

where $\textbf{u}= (u,v)$ is the local fluid velocity vector.

The conservation of mass is described by the continuity equation

8 $$\begin{aligned} \partial _t \rho + \nabla \cdot (\rho {\textbf {u}})= 0. \end{aligned}$$

On the other hand, the conservation of momentum is governed by the Navier–Stokes equations:

9 $$\begin{aligned}{} & {} \partial _t (\rho \textbf{u}) + \nabla \cdot (\rho \textbf{u} \textbf{u})\nonumber \\{} & {} \quad = \!-\! \nabla p \!+\! \nabla \cdot \left[ \eta \left( \nabla \textbf{u} \!+ \!\nabla \textbf{u}^T \!-\! \frac{2}{3} (\nabla \cdot \textbf{u}) \textbf{I}\right) \right] \! -\! \phi \nabla \mu . \end{aligned}$$

where $\eta$ is the dynamic viscosity, $\textbf{I}$ is the identity matrix, and *T* denotes a transpose. Here, the pressure obeys $p=\rho /3$ and follows from the ideal-gas term in Eq. (1). The last term in Eq. (9) is the contribution to the conservation of momentum arising from variations in the chemical potential. This term leads to capillary stresses in the interfacial region, but also to an osmotic-type force in the bulk of the fluid phases. Even though Eqs. (8) and (9) allow for variations in the mass density, the typical Mach numbers in the simulations are of order $Ma\approx 10^{-8}$. Therefore, effects due to compressibility are negligible.

In summary, the governing equations comprise the Cahn–Hilliard equation for the order parameter $\phi (\textbf{r},t)$ and the continuity and Navier–Stokes equations for the fluid velocity $\textbf{u}(\textbf{r},t)$, Eqs. (7), (8), and (9). These are complemented by boundary conditions at the solid walls and at the edges of the domain (see supplementary information).

At the solid boundaries, located at $y=0$ and $y=H$ for $0\le x\le L$, we implement the boundary conditions [25, 26]

10 $$\begin{aligned}{} & {} \partial _n\phi =-\sqrt{8}\chi /3\gamma _{\textrm{eq}} l, \end{aligned}$$

11 $$\begin{aligned}{} & {} \partial _n \mu =0, \end{aligned}$$

12 $$\begin{aligned}{} & {} \textbf{u}=0, \end{aligned}$$

and

13 $$\begin{aligned} \partial _n p=0. \end{aligned}$$

Equations (10) and (11) introduce wettability and no-flux conditions at the solid. Equations (12) and (13) impose stick and impenetrability conditions at the solid boundaries. It is well-known that the Cahn–Hilliard equation, coupled to the Navier–Stokes equations, gives rise to a slip effect which allows the motion of the contact line. This effect has been shown to match the hydrodynamic description of contact-line dynamics as given by the Cox–Voinov theory [27].

At the side edges of the domain, located at $x=0$ and $x=L$ for $0\le y \le H$, we implement the boundary conditions [26, 28]

14 $$\begin{aligned}{} & {} \phi =\phi _i, \end{aligned}$$

15 $$\begin{aligned}{} & {} \mu =\frac{3\gamma _{\textrm{eq}}}{\sqrt{8}l} \left( \phi _i^3 - \phi _i\right) , \end{aligned}$$

16 $$\begin{aligned}{} & {} \partial _n\textbf{u}=0, \end{aligned}$$

and

17 $$\begin{aligned} p=\frac{3\gamma _{\textrm{eq}}}{\sqrt{8}l} \left( \frac{3}{4}\phi _i^4 - \frac{1}{2}\phi _i^2\right) + \frac{\rho _0}{3}, \end{aligned}$$

where $\rho _0$ is the mean bulk density of the droplet. The purpose of Eqs. (14) and (15) is to induce a diffusive current that drives the phase change in the Cahn–Hilliard equation. Here, the boundary value $\phi _i$ is set independently at each edge, i.e. $\phi _i=\phi _{l}$ if $x=0$ and $\phi _i=\phi _r$ if $x=L$, with $\phi _l$ and $\phi _r$ chosen to drive the phase change [22]. Accordingly, the chemical potential is fixed by Eq. (15), which follows from Eq. (5) neglecting the contribution of the Laplacian term. To drive the evaporation of the droplet, it suffices to set $\phi _i<-1$, which implies $\mu <0$ in Eq. (15). To drive condensation, we use $-1<\phi _i<0$ and $\mu >0$. The boundary conditions in Eqs. (14) and (15) introduce a force density due to the last term in the Navier–Stokes equations. To compensate for this force, we impose the Dirichlet boundary condition on the pressure, Eq. (17).

### Simulation setup

**Table 1:** Model parameters used in the simulations in lattice-Boltzmann units

*L*	*H*	$\gamma _{\textrm{eq}}$	*l*	*M*	$\eta$	$\rho$
128	64	0.0001	1.6	18	1/6	1

**Table 2:** Boundary values used in the simulations

$\phi _l$	$\phi _r$	$\mu _l$	$\mu _r$	$\theta _e$
$-1.0$	$-1.6$	0	$-1.65\times 10^{-4}$	$90^\circ$
$-1.1$	$-1.5$	$-1.53\times 10^{-5}$	$-1.24\times 10^{-4}$	$90^\circ$
$-0.9$	−1.3	$1.13\times 10^{-5}$	$-5.95\times 10^{-5}$	$90^\circ$
−0.8	−1.1	$1.91\times 10^{-5}$	$-1.53\times 10^{-5}$	$90^\circ$
−0.7	−0.9	$2.37\times 10^{-5}$	$1.13\times 10^{-5}$	$90^\circ$
$-1.0$	$-1.6$	0	$-1.65\times 10^{-4}$	$60^\circ$
−1.0	$-1.6$	0	$-1.65\times 10^{-4}$	$80^\circ$
−1.0	−1.6	0	$-1.65\times 10^{-4}$	$100^\circ$
$-1.0$	$-1.6$	0	$-1.65\times 10^{-4}$	$120^\circ$

To integrate the model equations, we use a lattice-Boltzmann algorithm as detailed in reference [22]. We set an initial condition consisting of a droplet of initial base radius $R_0$ and contact angle $\theta _e$ positioned at the centre of the simulation domain, as shown in Fig. 1. The order parameter within the droplet is set to $\phi =1$ and in the ambient gas phase to $\phi =-1$. The fluid velocity is set to $\textbf{u}=0$ across the whole of the simulation domain. Simulations are run for typically $3\times 10^6$ simulation steps. The rest of the fluid properties used are summarised in Tables 1 and 2.

For a given choice of the boundary values of the chemical potential, $\mu _l$ and $\mu _r$, the characteristic time of diffusive transport across the simulation domain, $t_c$, follows from Eq. (7) and reads

18 $$\begin{aligned} t_c = \frac{L^2}{M|\mu _l-\mu _r|}. \end{aligned}$$

In the following, we report our results using *L* and $t_c$ as the characteristic length and time scales.

## Results

**Fig. 2:**
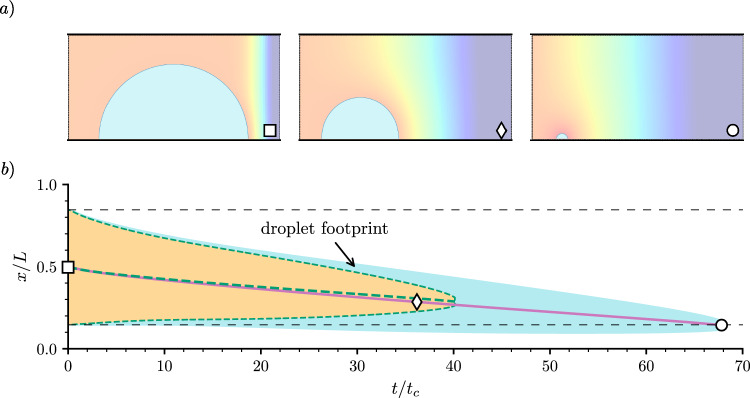
Droplet evaporation under a chemical potential gradient. **a** Evaporation sequence of a droplet with $\theta _e=90^\circ$ subject to a gradient in the ambient chemical potential, where $\mu _l=0$ and $\mu _r=-1.7\times 10^{-4}$. **b** Evolution of the *x*-coordinate droplet’s centre (solid purple line) and its footprint (blue shaded area). The empty symbols correspond to the snapshots of panel (**a**). The horizontal thin dashed lines indicate the initial position of the left and right droplet edges. The orange shaded area encased in green dashed lines shows the evolution of a droplet subject to a higher evaporation rate, with $\mu _l=-1.5\times 10^{-5}$ and $\mu _l=-1.2\times 10^{-4}$

Figure 2a shows the evolution of a droplet of initial radius $R_0=0.35 L$ and equilibrium contact angle $\theta _e=90^\circ$ subject to the boundary conditions $\mu _l=0$ and $\mu _r=-1.7\times 10^{-4}$. The chemical potential field is shown as a colour map and varies continuously between the edges of the domain across the gas phase. The resulting gradient induces evaporation from the right-hand side of the droplet, while the left-hand side is kept close to equilibrium. As the droplet evaporates, its centre moves towards the left of the domain, i.e. towards the region of weaker evaporation. Figure 2b shows the position of the droplet’s centre (solid purple line) and its footprint, which corresponds to the contact length with the solid (blue shaded area). In the apparent absence of condensation, it is clear both edges move to the region of lower evaporation, confirming that the difference in chemical potential induces a propulsion mechanism.

The same effect can be observed for a variety of combinations of the boundary values $\mu _l$ and $\mu _r$, and equilibrium contact angle, $\theta _e$, which we report in Table 2. In all cases, the droplet exhibits motion towards the side where the vapour concentration in the gas phase is higher. For evaporation, this corresponds to the region of weaker evaporation rate. For condensation, this is the region where the condensation rate is higher. For example, setting $\mu _l=-1.5\times 10^{-5}$ and $\mu _r=-1.2\times 10^{-4}$, i.e. driving evaporation from both sides, but at a stronger rate on the right-hand side, induces droplet motion from right to left, indicated by the yellow area in Fig. 2b.

**Fig. 3:**
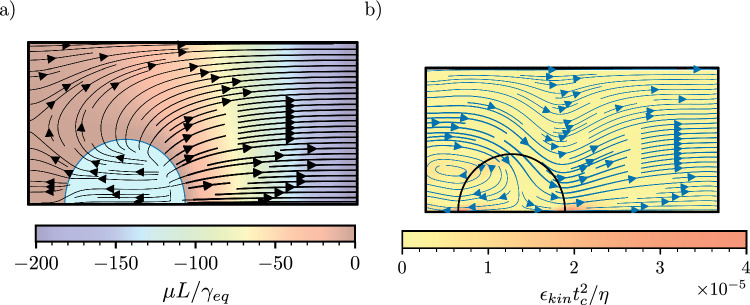
Mass flux and flow pattern during droplet evaporation. **a** Diffusive flux (stream plot) and chemical potential (colour map). **b** Velocity field (stream plot) and viscous dissipation of energy (colour map). The simulation parameters are as in Fig. 2a at time $t/t_c = 36$

Figure 3a shows a stream plot of the order-parameter flux, $\textbf{J}$, for the same simulation parameters of Fig. 2a, where the chemical potential values are $\mu _l=0$ and $\mu _r=-1.7\times 10^{-4}$. The flux varies along the interface, growing from left to right, but is always directed from the interface into the gas phase. This rules out accumulation of mass in the region of weaker evaporation side as the mechanism leading to droplet motion. The flow pattern, shown in Fig. 3b, shows an overall flow from left to right in the gas phase. This is expected from the Cahn–Hilliard equation, where a diffusive flux can also trigger a flow due to advection. We also observe a flow within the droplet from its centre-top towards the edges, which is consistent with a stronger evaporation rate at the contact points. To characterise the local friction associated with the flow, we compute the rate of viscous dissipation of energy density [29],

19 $$\begin{aligned} \epsilon _{kin} = 2 \eta \left[ (\partial _xu)^2+(\partial _yv)^2+\frac{1}{2}\left( \partial _yu+\partial _xv\right) ^2\right] , \end{aligned}$$

shown as a colour map in Fig. 3b. The motion of the contact lines creates regions of higher dissipation at the edges of the droplet, with a stronger effect on the right edge, where evaporation is stronger. This observation suggests that the droplet motion mechanism is hydrodynamic in nature, as it leads to motion of the interface past the solid surface.

Based on these observations, we expect that droplet motion can be influenced by two different driving mechanisms. The first is due to a Marangoni flow, which drives motion towards regions where the droplet-gas surface tension is higher [11]. The second mechanism is the capillary force due to a difference in wettability of the surface, favouring motion towards regions of higher surface energy. This force can be expressed as the unbalanced Young stress, $F_x=(\gamma \cos \theta )_r-(\gamma \cos \theta )_l$, where *r* and *l* refer to the right and left contact points, respectively. Note that, in general, both the surface tension, $\gamma$, and the contact angle, $\theta$, can vary between the droplet’s edges.

In the Cahn–Hilliard model the surface tension depends on the local composition of the binary fluid. Such a dependence implies that the surface tension varies along the droplet interface if the composition of the surrounding gas phase also varies. To measure the surface tension along the interface of the droplet, we first define the arc length, *s*, which runs from the left to the right edge along the interface, as shown in Fig. 1. At each point of the interface, we compute the normal coordinate, *n*, and then calculate the surface tension as the excess free energy [30]

20 $$\begin{aligned} \gamma = \int _{n_\textrm{min}}^{n_\textrm{max}} [\psi (\phi ,\nabla \phi ) -\psi (\phi _{\textrm{eq}},0)] \textrm{d} n. \end{aligned}$$

**Fig. 4:**
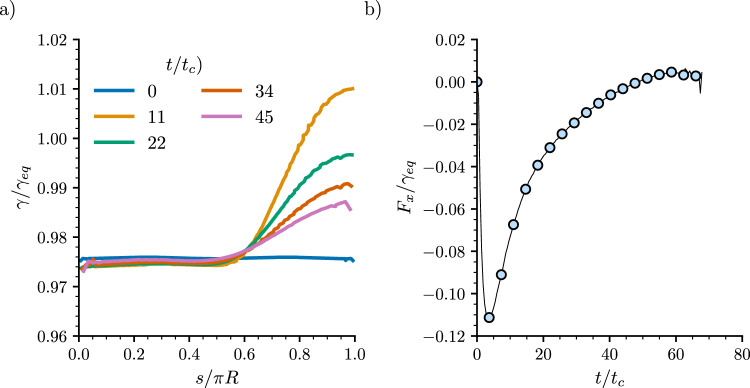
Driving forces acting on a droplet under a chemical potential gradient. **a** Surface tension profile as a function of the arc length along the droplet’s surface. The arc length is measured from the left to the right edges (see Fig. 1). **b** Total Young’s stress, $F_x$, as a function of time. Negative values correspond to a net force acting in the direction of motion of the droplet. Simulation parameters are as in Fig. 2a

Figure 4a shows $\gamma (s)$ profiles at different times for the simulation parameters of Fig. 2a, i.e. $\mu _l=0$ and $\mu _r=-1.7\times 10^{-4}$. Overall, $\gamma$ increases from left to right, i.e. towards the region of higher evaporation rate. In the model, evaporation is driven by imposing the boundary value $\phi _r<-\phi _{\textrm{eq}}$, which implies a lower concentration of the liquid component in the gas-rich phase. Therefore, it is reasonable that the surface tension is higher in this region. The variation of the surface tension is expected to generate a Marangoni flow from left to right, in the direction of the arc-length. As shown in Fig. 3b, the flow pattern within the droplet shows this asymmetry, with flow predominantly occurring towards the region of higher surface tension.

Figure 4b shows the unbalanced Young stress, $F_x=(\gamma \cos \theta )_r-(\gamma \cos \theta )_l$, as a function of time. While the surface tension at the right edge of the droplet is larger than at the left, the contact angle shows the opposite trend, i.e. it is higher on the left edge of the droplet. The overall result is a negative force, pointing to the left, in the same direction of droplet motion. The variation of $F_x$ in time agrees well with the motion of the droplet, decreasing with time until the droplet comes to rest at long times. These direct measurements support that the variation of the evaporation rate along the interface induces a net capillary force, driving the droplet towards regions of lower evaporation. Since there are no chemical potential gradients inside the droplet, the Young stresses that arise across the droplet footprint are only influenced by the concentration difference in the vapour phase; therefore, large chemical potential gradients occur on the side of the gas phase against the solid and the fluid. From the results we conclude that, since the liquid–gas inhomogeneity is pointing against the direction of motion of the droplet, it is the solid–liquid surface tension inhomogeneity which dominates the dynamics of the droplet. In regard to a variation of the equilibrium contact angle, we found that different surface wettabilities consistently lead to motion towards lower regions of evaporation. However, a rigorous measurement of the unbalanced Young stress in such cases is not possible. This is due to the diffuse nature of the interface in the simulations, which makes it difficult to extract an accurate measurement of the surface tension close to the solid wall as the contact angle deviates from $90^\circ$.

We now turn our attention to the results obtained for the chemical potential values presented in Table 2, which, for brevity, we include in the supplementary information. The results show a consistent motion of the centre of mass of the droplet towards more humid regions for all the cases explored. However, there are two considerations for the results presented in this study. When the chemical potential at the boundaries is a combination that results in condensation on one side of the droplet and evaporation on the other, or condensation on both sides, the driving force for the motion of the droplet is difficult to measure. This is because the gain and loss of mass on each side of the droplet leads to a kinematic effect that masks the effect of the driving force. On the other hand, when the droplet undergoes condensation on both sides, the range of boundary values for the chemical potential is limited by the spontaneous adsorption of the condensing phase on the solid surface. For the boundary values tested, there is an overall motion of the centre of mass of the droplet towards more humid regions, and this suggests that the same mechanism leading to the motion of the droplet reported in Fig. 2, $\mu _l=0$ and $\mu _r=-1.7\times 10^{-4}$, is also at play for the different boundary values of the chemical potential.

## Discussion and conclusions

In this work we have studied the motion of a droplet in the presence of a gradient in the ambient composition, here modelled using the Cahn–Hilliard diffuse-interface model. This situation is similar to the evaporation or condensation of a single-component droplet driven by a gradient of concentration of the vapour in the gas phase. In the simulations, we have identified two main capillary effects caused by the composition of the gas phase. First, we have identified an imbalance of the Young’s stress at the edges of the droplet, which points towards regions where the gas phase has a higher concentration of vapour. On the other hand, the surface tension increases towards regions where the gas has a lower vapour concentration. The effect of the unbalanced Young’s stress implies a higher wettability of the surface in regions of higher vapour concentration. Such an effect dominates the dynamics, as the droplet consistently migrates in the same direction.

Previous studies have focused on the vapour-mediated interaction of pairs of droplets undergoing evaporation. Man and Doi [19] studied the evaporation of pairs of droplets of a single liquid component using a one-sided sharp-interface model based on lubrication theory. The effect of a non-uniform composition in the gas phase was modelled by introducing gradient in the evaporation rate at the interface, thus affecting the droplet’s shape locally. Their model predicts droplet motion to regions of lower evaporation rate, which they ascribe to the tendency of the droplet to reduce viscous energy dissipation by adjusting the portion of the interface that evaporates faster to maintain a close-to-equilibrium shape. Wen et al. [17] carried out experiments of hexane droplets evaporating on high-energy glass surfaces and reported motion towards regions of higher vapour concentration in the gas. Sadafi al. [18] demonstrated that evaporative cooling can induce a temperature gradient and, consequently, a thermal Marangoni flow.

While a gradient in relative humidity in the gas phase leads to the expected gradient in evaporative flux across the interface and to a higher viscous energy dissipation at the contact edges of the droplet as proposed in Ref. [19]; this work additionally demonstrates the further presence of a sorptive Marangoni flow driven by the local concentration of vapour in the gas phase. Importantly, this effect drives a flow towards regions of high evaporation, in contrast to the solutal and thermocapillary flows reported in Ref [18].

The main aim of this work is to understand the effect of concentration gradients in the vapour phase on a droplet undergoing a phase change on a solid surface. Here, we have focused on a simple 2D geometry and have neglected the effect of temperature variations arising from the phase change. Extending this work to consider 3D droplets is interesting, as gradients could potentially allow a stronger propulsion as the variation of the surface tension would occur over a larger area. Investigating the effect of a temperature gradient coupled with the effect of a concentration gradient would provide a better understanding of the importance of one relative to the other in determining the droplet motion.. From the experimental perspective, there are two aspects that play a crucial role in the potential study of this effect in a laboratory setting. The first one is the ability to control the temperature changes involved in the system, for instance, by using low volatile liquids in a temperature-controlled humidity chamber. The second aspect would be to work on ultra-smooth surfaces, where surface tension contributions are significant enough to contribute on the dynamics of the surface, which can be achieved employing surfaces that exhibit low friction [31–33]. We hope that this work serves as motivation to continue looking into this effect in future studies.

### Supplementary Information

Below is the link to the electronic supplementary material.

Supplementary file 1 (pdf 777 KB)

## Data Availability

Datasets generated during the current study are available from the corresponding author on reasonable request.
