# Supplementary material for: Droplet motion driven by humidity gradients during evaporation and condensation
Source: Eur Phys J E Soft Matter. 2024 May 13;47(5):32. doi: 10.1140/epje/s10189-024-00426-7 (PMC11089009; doi:10.1140/epje/s10189-024-00426-7)
Supplement: Supplementary file 1 — (pdf 777 KB) [file 10189_2024_426_MOESM1_ESM.pdf]

Self-propulsion of droplets driven by humidity gradients during evaporation and condensation: Supplementary information

Hernán Barrio-Zhang,[1,*] Élfego Ruiz-Gutiérrez,[2] Daniel Orejon,[1] Gary G. Wells,[1] and Rodrigo Ledesma-Aguilar[1]

[1]*Institute for Multiscale Thermofluids, School of Engineering, University of Edinburgh, The King's Buildings, Mayfield Road, Edinburgh EH9 3FB, United Kingdom*

[2]*School of Engineering, Newcastle University, Claremont Road, Newcastle upon Tyne, NE1 7RU, United Kingdom*

(Dated: March 15, 2024)

* hbarrio@ed.ac.uk

I. LATTICE BOLTZMAN ALGORITHM

A. General method

In order to numerically solve the equations of state under this framework, we make use of the Lattice Boltzmann method, a solver which considers both macroscopic and microscopic interactions. The Boltzmann equation, which arises from the Kinetic Theory of Gases [1], is a statistical description of the evolution of a fluid. The discretised form of the Boltzmann equation is what is referred to a the Lattice Boltzmann equation [2, 3] To perform the Lattice Boltzmann method, a distribution function is defined, $f_q(x)$, that corresponds to the mean density of particles found at an instance in time t in a discrete position x and a velocity c_q [4].

The Lattice-Boltzmann Method consists of two main steps [2, 4, 5], the first one is called the streaming step which describes the mean density of particles in a given position and velocity at a certain time and is given by the distribution function

$$f_q(x + c_q, t + 1) = f_q^\star(x, t). \quad (1)$$

The second step is called the collision step, which along post-collision terms, describes how the distribution of particles has changed due to interactions in the streaming step. The distribution function associated with the collision step is expressed as follows

$$f_q^\star(x, t) = f_q(x, t) + \Xi[f]_q + S_q, \quad (2)$$

where $\Xi[f]_q$ is the collision operator and S_q is the sources term.

During the streaming step, the particle populations interact with neighboring lattice nodes by means of the velocity c_q over a unitary time step such that $x \to x+c_q$. This defines a set of velocities associated with neighboring lattice points (depending on the dimension of the domain) in the form of $c_q \in \{c_q\}_{q=0}^{Q-1}$. The result of this interaction are a Q, D-dimensional integer vectors that describe the connectivity between the lattice points. This is synthesized by the Q-D notation in the Lattice Boltzmann model. For the case presented in this work, we use a $D2Q9$ in the Lattice Boltzmann notation, which implies a two-dimensional system with a connectivity of 3x3 neighbourhood.

On one hand, the collision operator in Equation 2 is defined using the BGK, where a local equilibrium distribution function, f_q^e, is defined. This function defines different relaxation rates for each component of the distribution function. The collision term bears a significant amount of importance in physical terms since it enables the user to tune transport coefficients such as the viscosity [4].

The source term in Equation 2, on the other hand, can take many forms which depend on the type of sources the user wants to be considered in the simulation, for instance, defining sources of mass, forces, or stresses.

From the Navier-Stokes equation, it is possible to obtain macroscopic parameters such as the density and the velocity. By using the Lattice Boltzmann Method to numerically integrate the Navier-Stokes equations, one can define the expression of macroscopic variables in terms of the distribution function [4]. The mass density flux density yields

$$\rho = \sum_{q=0}^{Q-1} f_q, \tag{3}$$

and the momentum of the density, from where one can obtain the velocity field, is defined as [6]

$$\rho u = \sum_{q=0}^{Q-1} c_q f_q, \tag{4}$$

The Cahn-Hilliard equation allows us to obtain the value of the phase field. In order to compute it, a second distribution function, g_q, of similar form as Equations 1 and 2 is used where the moments and collision parameters differ from f_q. The zeroth moment of this distribution function defines the phase field as [6]

$$\phi = \sum_{q=0}^{Q-1} g_q, \tag{5}$$

To calculate the values of the pressure tensor field and the chemical potential, it is necessary to obtain the gradient and the Laplacian of the phase field by a 3x3 finite differences stencil. These can be approximated by using the finite difference stencils, which for the gradient yields

$$\nabla \phi \approx \frac{1}{c_s^2} \sum_{q \neq 0} w_q c_q \phi(x + c_q), \tag{6}$$

and for the Laplacian

$$\nabla^2\phi \approx \frac{2}{c_s^2}\sum_{q\neq 0} w_q[\phi(x+c_q)-\phi(x)], \tag{7}$$

where w_q is used as weighting factors that optimise the accuracy of the approximation [7].

B. Boundary conditions of the lattice-Boltzmann algorithm

The boundary conditions for the numerical method are such to give the correct representation of the diffuse interface system. For the solid boundary conditions, we implement an interpolated bounceback algorithm [8],

$$f_{\bar{q}}(\mathbf{x}, t+\Delta t) = f_q^{\star}(\mathbf{x}, t) \tag{8}$$

for the unknown particle population $f_{\bar{q}}$ travelling in the opposite direction to the solid surface, that is, $\bar{q}$ is such that $\mathbf{c}_{\bar{q}}+\mathbf{c}_q = 0$. The distance fraction, δ_q, is such that $\mathbf{x}_{\mathrm{b}} = \mathbf{x}+\delta_q\mathbf{c}_q$, where $\mathbf{x}_{\mathrm{b}}$ is the position of the solid surface. In Equation (8) $f_q^{\star}$ corresponds to the pre-streaming particle population, i.e., before updating the populations to $t+1$. This ensures that the velocity of the boundary is as in Eq. (12) of the manuscript and conserves mass density as in Eq. (13) of the main text. Similarly,

$$g_{\bar{q}}(\mathbf{x}, t+1) = g_q^{\star}(\mathbf{x}, t) \tag{9}$$

ensures that the phase-field is conserved and no diffusive fluxes permeate the solid boundaries.

At the open boundary, we implement an anti-bounceback algorithm [9]. Then,

$$f_{\bar{q}}(\mathbf{x}, t+1) = \frac{1}{2}\left[f_{\bar{q}}^{e}(\rho_i, \mathbf{u}_i) + f_q^{e}(\rho_i, \mathbf{u}_i)\right], \tag{10}$$

which satisfies Equation (17) of the main text and Equation (16) by extrapolating the velocity at the boundary, $\mathbf{u}_i = \mathbf{u}(\mathbf{x})$, which is assumed to be at $\mathbf{x}+\mathbf{c}_q(1/2)$. Similarly,

$$g_{\bar{q}}(\mathbf{x}, t+1) = \frac{1}{2}\left[g_{\bar{q}}^{e}(\phi_i, \mathbf{u}_i, \mu_i) + g_q^{e}(\phi_i, \mathbf{u}_i, \mu_i)\right], \tag{11}$$

which imposes the value of the phase field and chemical potential at the boundaries as in Eq. (14) and (15) of the manuscript, respectively.

For further details and validation of the numerical method and the different boundary conditions see Ref. [10].

II. ADDITIONAL SIMULATION RESULTS

A. Chemical potential gradients

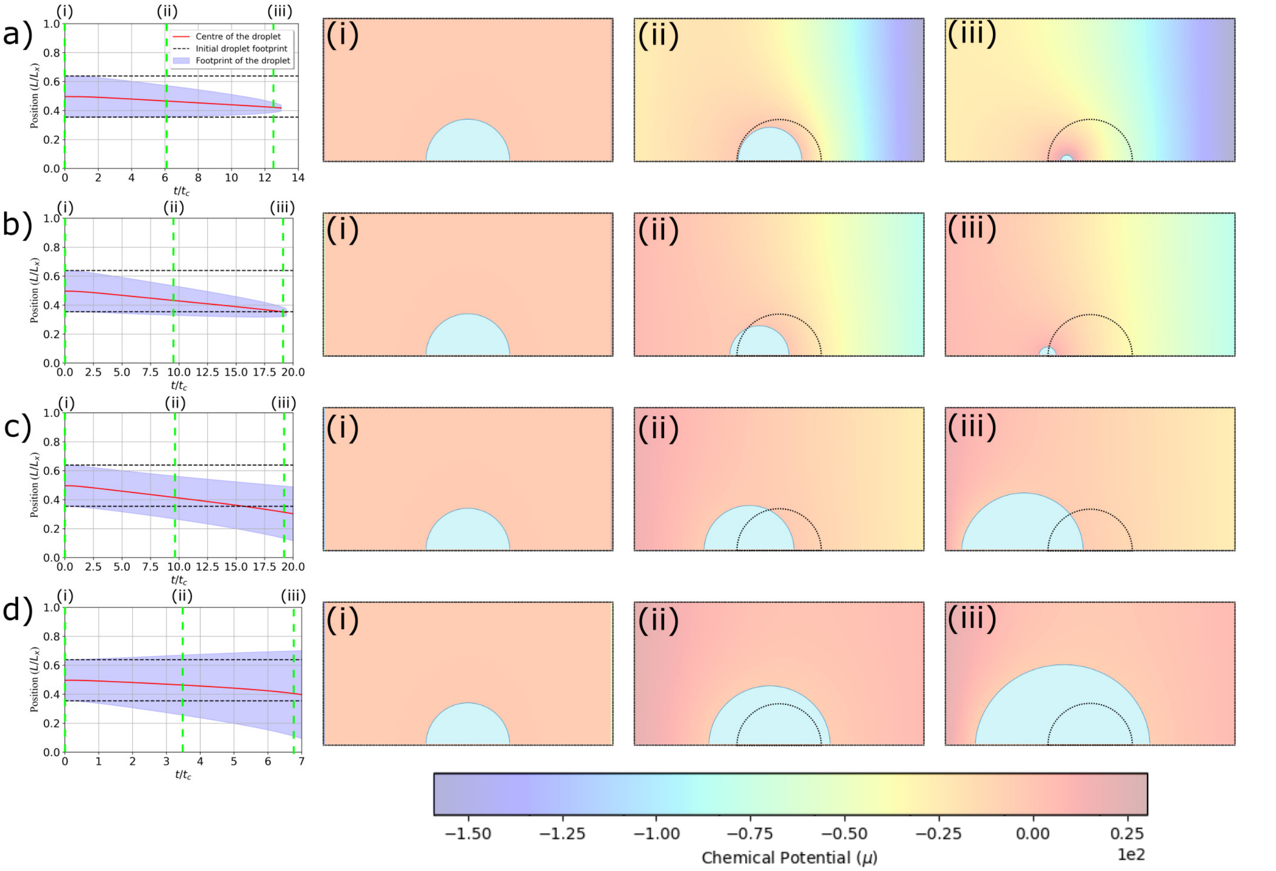

FIG. 1: **Chemical gradient regimes.** Figure showcasing the evolution of the droplet at different chemical potential gradients, where (i), (ii) and (iii) correspond to snapshots in the lifetime of the droplet. a) Evaporation-evaporation gradient where $\mu_l = 1.5 \times 10^{-5}$ and $\mu_r = -1.2 \times 10^{-4}$. b) Slight condensation and strong evaporation, where $\mu_l = 1.13 \times 10^{-5}$ and $\mu_r = -5.9 \times 10^{-5}$. c) Evaporation and condensation at a similar rate, where $\mu_l = 1.9 \times 10^{-5}$ and $\mu_r = -1.5 \times 10^{-5}$. d) Condensation-Condensation gradient, where $\mu_l = 2.4 \times 10^{-5}$ and $\mu_r = 0.6 \times 10^{-5}$. All initial radii are $R = 0.2$ and the colorbar for the chemical potential has been normalized by $\gamma_0/\rho L$.

B. Surface tension profile for condensation-condensation gradient

Figure 2 shows the surface tension profile of the strongest condensation gradient we explored. The change in surface tension along the interface of the droplet is negligible up to the point where the droplet approaches the boundary, generating stronger gradients due to the imbalance caused by the boundary conditions. Before this point, the apparent motion of the droplet is predominantly dominated by the phase change, as seen in Figure 1d).

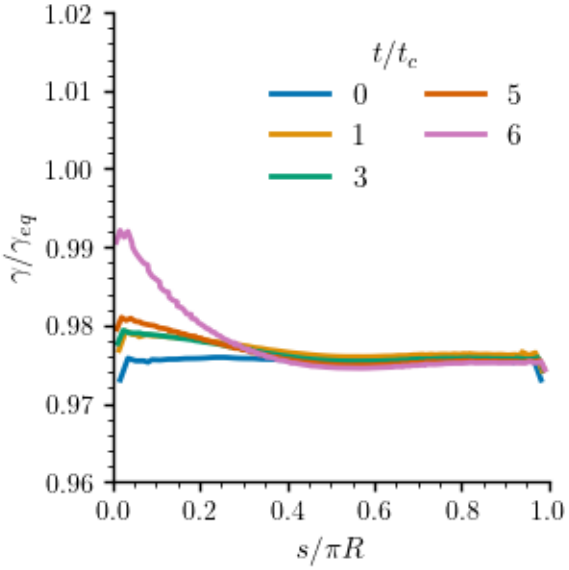

FIG. 2: **Surface tension profile for condensation-condensation regime**: For $\mu_l = 2.4 \times 10^{-5}$ and $\mu_r = 0.6 \times 10^{-5}$

C. Wettability limited surface tension profiles

Figure 3 shows a representation of the limitations of measuring surface tension for wetabilities that defer from neutral wetting. For hydrophobic regimes in row a) of Figure 3, a significant part of the gas phase is not captured. Analogously, for hydrophilic regimes, row b) of Figure 3, significant part of the liquid phase is not captured. This is due to the diffuse nature of the interface and leads to inaccurate measurements of the surface tension close to the contact points.

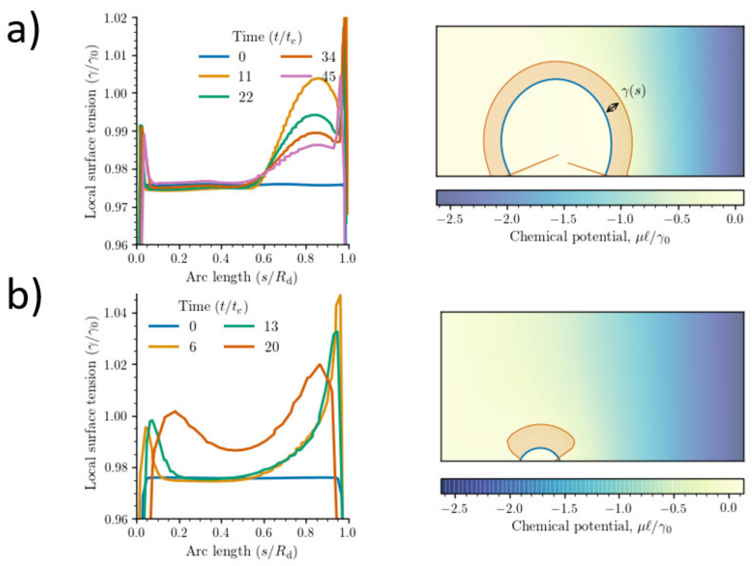

FIG. 3: **Surface tension profile for condensation-condensation regime**: For $\mu_l = 2.4 \times 10^{-5}$ and $\mu_r = 0.6 \times 10^{-5}$

REFERENCES

---

[1] R. L. Liboff, *Kinetic Theory.* Springer, third ed., 1983.

[2] T. Kruger, H. Kusumaatmaja, A. Kuzmin, O. Shardt, G. Silva, and E. M. Viggen, *The Lattice Boltzmann Method: Principles and Practice.* Springer, first edit ed., 2016.

[3] X. He and L. S. Luo, "A priori derivation of the lattice Boltzmann equation," *Physical Review E*, vol. 55, p. R6333, 6 1997.

[4] E. Ruiz-Gutiérrez, A. M. Edwards, G. McHale, M. I. Newton, G. G. Wells, C. V. Brown, and R. Ledesma-Aguilar, "Lattice Boltzmann Simulations of Multiphase Dielectric Fluids," *Langmuir*, vol. 37, pp. 7328–7340, 6 2021.

[5] R. Ledesma-Aguilar, D. Vella, and J. M. Yeomans, "Lattice-Boltzmann simulations of droplet evaporation," *Soft Matter*, vol. 10, pp. 8267–8275, 11 2014.

[6] E. Ruiz-Gutiérrez, J. H. Guan, B. Xu, G. McHale, G. G. Wells, and R. Ledesma-Aguilar, "Energy Invariance in Capillary Systems," *Physical Review Letters*, vol. 118, no. 21, 2017.

[7] C. M. Pooley and K. Furtado, "Eliminating spurious velocities in the free-energy lattice Boltzmann method," *Physical Review E - Statistical, Nonlinear, and Soft Matter Physics*, vol. 77,

no. 4, 2008.

[8] D. Yu, R. Mei, L.-S. Luo, and W. Shyy, “Viscous flow computations with the method of lattice Boltzmann equation,” *Progress in Aerospace Sciences*, vol. 39, pp. 329–367, 2003.

[9] I. Ginzburg, F. Verhaeghe, and D. dHumì eres, “Study of simple hydrodynamic solutions with the two-relaxation-times lattice boltzmann scheme Two-Relaxation-Time Lattice Boltzmann Scheme: About Parametrization, Velocity, Pressure and Mixed Boundary Conditions,” *COMMUNICATIONS IN COMPUTATIONAL PHYSICS*, vol. 3, no. 2, pp. 427–478, 2008.

[10] E. R. Gutierrez, *PhD thesis: Theoretical and computational modelling of wetting phenomena in smooth geometries.* PhD thesis, Northumbria University, 2017.
